# 3D-PAST: Risk Assessment Model for Predicting Venous Thromboembolism in COVID-19

**DOI:** 10.3390/jcm11143949

**Published:** 2022-07-07

**Authors:** Yi Lee, Qasim Jehangir, Chun-Hui Lin, Pin Li, Anupam A. Sule, Laila Poisson, Venugopal Balijepally, Abdul R. Halabi, Kiritkumar Patel, Geetha Krishnamoorthy, Girish B. Nair

**Affiliations:** 1 Department of Medicine, St. Joseph Mercy Oakland Hospital, Pontiac, MI 48341, USA; qasimjehangir@gmail.com (Q.J.); anupam.a.sule@stjoeshealth.org (A.A.S.); geetha.krishnamoorthy@stjoeshealth.org (G.K.); 2 Department of Public Health Sciences, Henry Ford Health System, Detroit, MI 48202, USA; clin4@hfhs.org (C.-H.L.); pli3@hfhs.org (P.L.); lpoisso1@hfhs.org (L.P.); 3 Department of Informatics, St. Joseph Mercy Oakland Hospital, Pontiac, MI 48341, USA; 4 School of Business Administration, Oakland University, Rochester, MI 48307, USA; balijepa@oakland.edu; 5 Division of Cardiology, St. Joseph Mercy Oakland Hospital, Pontiac, MI 48341, USA; arhalabi@hotmail.com (A.R.H.); kiritpmd@gmail.com (K.P.); 6 William Beaumont School of Medicine, Oakland University, Auburn Hills, MI 48307, USA; girish.nair@beaumont.org; 7 Division of Pulmonary and Critical Care Medicine, Beaumont Health System, Royal Oak, MI 48183, USA

**Keywords:** venous thromboembolism, SARS-CoV-2, COVID-19, risk assessment model, deep vein thrombosis, pulmonary embolism

## Abstract

Hypercoagulability is a recognized feature in SARS-CoV-2 infection. There exists a need for a dedicated risk assessment model (RAM) that can risk-stratify hospitalized COVID-19 patients for venous thromboembolism (VTE) and guide anticoagulation. We aimed to build a simple clinical model to predict VTE in COVID-19 patients. This large-cohort, retrospective study included adult patients admitted to four hospitals with PCR-confirmed SARS-CoV-2 infection. Model training was performed on 3531 patients hospitalized between March and December 2020 and validated on 2508 patients hospitalized between January and September 2021. Diagnosis of VTE was defined as acute deep vein thrombosis (DVT) or pulmonary embolism (PE). The novel RAM was based on commonly available parameters at hospital admission. LASSO regression and logistic regression were performed, risk scores were assigned to the significant variables, and cutoffs were derived. Seven variables with assigned scores were delineated as: **D**VT History = 2; High **D**-Dimer (>500–2000 ng/mL) = 2; Very High **D**-Dimer (>2000 ng/mL) = 5; **P**E History = 2; Low **A**lbumin (<3.5 g/dL) = 1; **S**ystolic Blood Pressure <120 mmHg = 1, **T**achycardia (heart rate >100 bpm) = 1. The model had a sensitivity of 83% and specificity of 53%. This simple, robust clinical tool can help individualize thromboprophylaxis for COVID-19 patients based on their VTE risk category.

## 1. Introduction

Thromboembolic complications are common in hospitalized patients with COVID-19. The frequency of venous thromboembolism (VTE) in hospitalized COVID-19 patients significantly varies from 1.7% to as high as 30% [1,2,3,4]. Along with large-vessel thrombosis, platelet–fibrin thrombi in small arterial vessels consistent with coagulopathy are also seen in the vast majority of patients who die from COVID-19 [5,6]. The possible mechanisms of VTE in COVID-19 include vascular endothelial inflammation and dysfunction caused by direct SARS-CoV2 viral infection, interference with the renin–angiotensin–aldosterone system, abnormal complement and coagulant pathway activation, abnormal platelet activation, and disseminated intravascular coagulation [7].

Studies have shown that VTE is associated with high mortality in COVID-19 patients [4,8]. Current guidelines support thromboprophylaxis in all hospitalized COVID-19 patients unless contradicted with the escalation of dosage in selected patients [9,10,11,12]. However, anticoagulation management remains heterogeneous across the world, given the varying levels of severity of illness, limited availability of confirmatory diagnostic imaging, and variabilities in the local hospital policies [13]. Despite the high incidence of VTE and the associated morbidity and mortality, there is no risk assessment model (RAM) dedicated to hospitalized COVID-19 patients to predict VTE. In this study, we sought to develop a robust, simplified RAM using simple variables such as presenting vitals, commonly tested lab metrics, and baseline comorbidities to help clinicians worldwide in their clinical decisions on VTE management in hospitalized COVID-19 patients.

## 2. Materials and Methods

### 2.1. Data Cleaning

**Figure 1:**
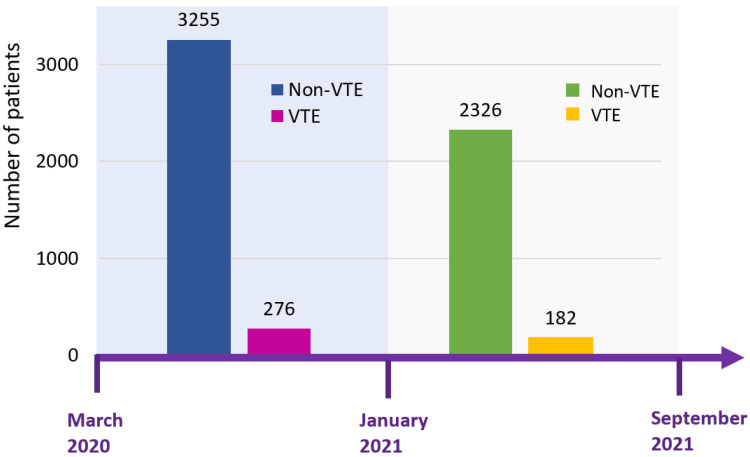
Timeline of two study cohorts of COVID-19. Patients from March to December 2020 were used for the training cohort, whereas patients from January to September 2021 were used for the validation cohort. The incidence of VTE was 7.8% in the derivation cohort and 7.3% in the validation cohort. Abbreviation: VTE, venous thromboembolism.

Data were included from one quaternary care and three community hospitals of the Henry Ford and Trinity Health systems. Clinical data were derived from electronic health records, deidentified, and stored in the Southeast Michigan COVID-19 Consortium Registry Database (SMCRD) using REDCap. As previously described [14], the SMCRD consists of data of patients who were hospitalized with a polymerase chain reaction (PCR)-confirmed SARS-CoV-2 infection. Each institution independently collected data both concurrently and retrospectively. This study was approved by the Trinity and Henry Ford health systems institutional review boards; the need for informed consent was waived for the use of deidentified patient data. Model training was performed on a cohort of patients hospitalized between 1 March and 31 December 2020, with validation performed on a cohort of patients hospitalized between 1 January and 5 September 2021 (Figure 1). Inclusion criteria were age ≥18 years and a positive SARS-CoV-2 PCR result. Collected data included presenting vital signs and laboratory values, baseline demographics, and past medical and social history abstracted using standard-text variables and International Classification of Diseases—Tenth Revision [ICD-10] codes (Supplementary Table S1).

The model was built to predict a composite outcome of in-hospital pulmonary embolism (PE) and deep vein thrombosis (DVT) as identified by standard-text variables and ICD-10 codes. Demographic variables included age, sex, race/ethnicity, and body mass index. Past medical history included hypertension, diabetes mellitus, hyperlipidemia, coronary artery disease, congestive heart failure, cerebrovascular accident, solid cancer and hematological malignancies, autoimmune disease, liver disease, lung disease, thyroid disease, atrial fibrillation, and prior history of DVT and PE. In social history, smoking and alcohol use were recorded. Vital signs including heart rate, respiratory rate, oxygen saturation, and systolic blood pressure were categorized. Laboratory values included complete blood count—white blood cell count, absolute lymphocyte count, absolute neutrophil count, neutrophil to lymphocyte ratio, platelet count; comprehensive metabolic panel—blood urea nitrogen, serum creatinine, total bilirubin, aspartate aminotransferase, alanine transaminase, alkaline phosphatase, serum albumin, serum potassium; cardiac disease-related biomarkers—B-type natriuretic peptide and troponin-I; and commonly tested markers for COVID-19 infection, including D-dimer, ferritin, C-reactive protein, lactate dehydrogenase, interleukin-6, lactate, and procalcitonin; the labs were also categorized (Supplementary Table S2).

### 2.2. Missing Data Handling

A total of 48.5% of patients had 90% of the data, and 73.4% of patients had 80% of the data. For demographics, social history, and vital variables, there were less than 5% missing data. For medical history variables, there were approximately 16% missing data. For laboratory values, the missing data rate ranged between 3.1% and 87.5%. Overall, a total of 48.5% of patients had 90% of the data, and 73.4% of patients had 80% of the data. The heat map demonstrating the missing data is shown in Supplementary Figure S1. Multivariate imputation by chained equations (MICE) was conducted to impute missing values for variables. We categorized presenting vital signs first before applying MICE. However, for laboratory values, we applied MICE first and then categorized the variables. An imputed dataset was derived by using predictive mean matching for numeric variables, logistic regression for binary variables, and Bayesian polytomous regression for factor variables.

### 2.3. Model Building

In the variable selection and model building, a total of 3531 hospitalized patients were included. A spectrum of variables (N = 48) was included in the selection process. We tested 2 different regression models, (1) Least Absolute Shrinkage and Selection Operator (LASSO) regression; (2) forward stepwise selection. LASSO regression was applied to handle potential collinearity and overfitting of variables. LASSO regression added an L1-penalized term in the conventional ordinary least square loss function $argmin_{\beta }{\left(y-x\beta \right)}^{T}\left(y-x\beta \right)$ to avoid excessive variables selected with a tuning parameter, which controls the degree of penalty. The L1-penalized term $\lambda_{1}\overset{p}{\underset{j=1}{\sum }}\left|\beta_{j}\right|$ allows weaker factors to be shrunk to zero, thus including only the strongest predictors in the model. With the L1 penalty, LASSO not only helps in reducing overfitting but can help in feature selection. In LASSO, cross-validation was used to select the tuning parameter for the best model. On the other hand, in the forward selection, the Akaike information criterion (AIC) was used as a criterion to select the best model with the minimum AIC. After comparing three different models, the LASSO model was chosen as the final model with better performance (area under the receiver operating characteristic (AUROC), etc.) and interpretability.

### 2.4. Score Assigning

Variables chosen by LASSO regression were included in the logistic regression model. We generated a simplified score by assigning scores based on the significance of coefficients. Each patient with or without VTE received a score. The risk score cutoffs were then derived, and the model accuracy was assessed by sensitivity, specificity, positive predictive value, negative predictive value, and AUROC.

### 2.5. Bootstrapping and Validation

To assess the reproducibility of the model, we derived 500 bootstrap resamples from patients admitted between 1 March and 31 December 2020. To assess the generalizability of the model, validation was performed on another cohort of patients hospitalized between 1 January and 5 September 2021. The validation dataset was generated by the exact same process as the derived dataset. The risk score and its cutoff were calculated as described above. All statistical analysis was performed using R statistical software version 4.0.4 (R Project for Statistical Computing, Boston, MA, USA), and *p* < 0.05 was considered statistically significant.

## 3. Results

### 3.1. Patient Characteristics

A total of 3531 patients were included in building the scoring prediction model, and 2508 patients were used for validation. The demographic characteristics of the derivation and validation cohorts are summarized in Table 1, and Supplementary Table S3. In the deviation cohort, the mean age of the population was 67.4 ± 16.4 years, with 49.7% females. Meanwhile, in the validation cohort, the mean age of the population was 60.9 ± 17.9 years, with 52.7% females. The incidence of VTE was 7.8% in the derived cohort and 7.3% in the validation set.

### 3.2. Risk Assessment Model

**Figure 2:**
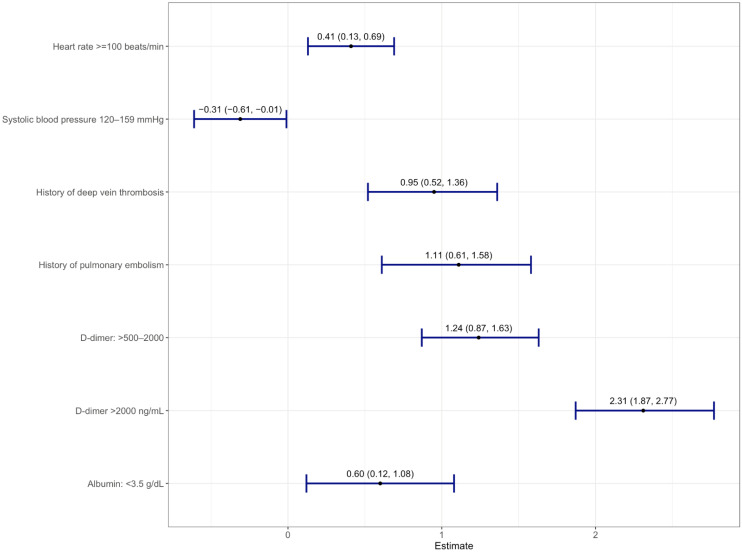
Forest plot of multivariable logistic regression showing seven variables with log-odds along with a 95% confidence interval from derivation cohort predictive of acute venous thromboembolism.

**Figure 3:**
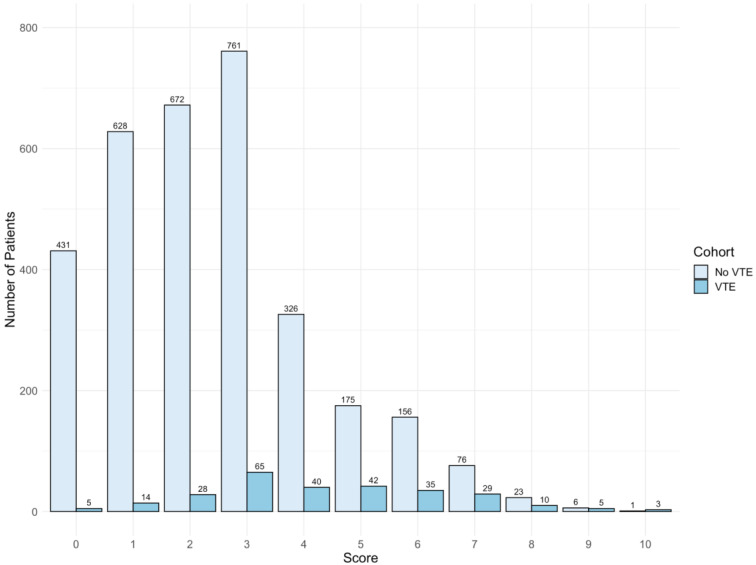
Histogram of risk assessment model score of venous thromboembolism (VTE) and non-VTE groups. The median score is 4 and 2 for VTE and non-VTE group, respectively. Overall, 49.7% scored below 3, whereas 50.4% of VTE patients scored 3 and above.

Thirty-four variables selected from the LASSO model were analyzed in a multivariate logistic regression model with VTE as the outcome (Supplementary Table S4). A total of seven variables were significantly associated with VTE (Figure 2). The scores assigned for each predictor are listed as follows: ***D***VT *H*istory = 2; High **D**-Dimer (>500–2000 ng/mL) = 2; Very *H*igh ***D***-Dimer (>2000 ng/mL) = 5; **P**E History = 2; Low **A**lbumin (<3.5 g/dL) = 1; **T**achycardia (heart rate >100 bpm) = 1; **S**ystolic Blood Pressure (<120 mmHg) = 1, denoted as “3D-PAST” for COVID-19-associated VTE (Table 2). Systolic blood pressure >120–159 mmHg was associated with lower risk for VTE (odds ratio 0.73; 95% confidence interval (CI): 0.54–0.99). Therefore, to assign a positive score to this variable, we used the systolic blood pressure <120 mmHg categories. The difference in risk between normal (90–119 mmHg) and low (<90 mmHg) systolic blood pressure was not statistically different (*p* = 0.06); hence, we merged normal and low into a single category (<120 mmHg) with +1 point. Patients with scores of 0 to 2 (*n* = 1778) had a lower risk of VTE (2.6%), whereas patients with a score of 3 or higher (*n* = 1753) had an increased risk of VTE (13.1%). The median scores were 4 and 2 for the VTE and non-VTE groups, respectively. A total of 49% of non-VTE patients scored below 3, whereas 51% of VTE patients had scores above 3 (Figure 3). The discrimination was assessed by a confusion matrix, which showed a sensitivity of 0.83, specificity of 0.53, positive predictive value (PPV) of 0.13, and negative predictive value (NPV) of 0.97. The AUROC was 0.751 (95% CI: 0.722–0.779, *p* < 0.05). The calibration of the RAM was good over the range of risk (Brier score 0.064). Increasing the cutoff of the model to 4 increased the specificity from 53% to 77% but at the cost of sensitivity (decreased from 83% to 59%). We chose a cutoff with a higher sensitivity than specificity (cutoff score 3) to assist the clinicians in identifying patients at increased risk of VTE (Supplementary Table S5).

### 3.3. Bootstrapping and Validation

In the bootstrapped sample, patients with scores of 0 to 2 (N = 237) had a VTE risk of 2.53%, whereas patients with scores of 3–10 (N = 263) had high a VTE risk of 15.6%. Bootstrapped sampling showed sensitivity of 0.87, specificity of 0.51, PPV of 0.16, NPV of 0.97, and AUROC of 0.73 (95% CI: 0.66–0.79) (Supplementary Figure S2). Similarly, in the validation data set, patients with scores of 0 to 2 (N = 1406) had a lower risk of VTE, at 3.2%, whereas patients with a score of 3 or higher (N = 1102) had an increased risk of VTE, at 12.4%. The validation cohort had sensitivity of 0.75, specificity of 0.59, PPV of 0.12, NPV of 0.97, and AUROC of 0.74 (95% CI: 0.70–0.77). The Brier score was 0.054 for the bootstrapped sample and 0.056 for the validation set, showing good calibration in the validation datasets.

### 3.4. Comparison to Sequential Organ Failure Assessment Score

The sequential organ failure assessment score (SOFA) score was recently shown to be a reliable tool for identifying COVID-19 patients at high risk for DVT [15]. We compared the SOFA score to our RAM for the risk stratification of VTE and validated it in our patient cohort. The AUROC of the SOFA score was 0.60 (95% CI: 0.57–0.63) (0.57 (95% CI: 0.53–0.61) for the derivation cohort and 0.63 (95% CI: 0.58–0.67) for the validation cohort) (Supplementary Figure S3). The results were inferior compared to the AUROC of 0.74 (95% CI: 0.70–0.77) of our RAM.

## 4. Discussion

Venous thromboembolism is a common complication of SARS-CoV-2 infection in hospitalized patients. However, there is a modest increase in major bleeding up to 4% in patients receiving full-dose anticoagulation [16,17]. The high incidence and worse outcomes associated with VTE highlight the need for a simple prediction model to identify individuals who are at increased risk of developing these thromboembolic events. Herein, we present a RAM with a sensitivity of 0.75, specificity of 0.59, and AUROC 0.74 in the validation cohort, which can be used in any clinical setting to predict the risk of acute VTE in COVID-19 patients.

There is a lack of consensus on the optimal dosage of anticoagulation in COVID-19 patients. Our model should assist clinicians in weighing the benefits of anticoagulation versus the risks of bleeding and help in deciding the initiation, and, more importantly, the continuation, of full-dose anticoagulation in the setting of increasing oxygen requirement. To the best of our knowledge, this is the first risk assessment scoring model dedicated to the inpatient COVID-19 population, with one prior model built for the cancer population admitted with COVID-19, with no validation on a separate dataset of patients [18]. This, along with other VTE studies that have validated the pre-COVID RAMs, included patients only from the early phase of pandemic [19,20,21].

In our large-cohort study that included a total of 6039 COVID-19 patients, we studied two waves of the COVID-19 pandemic in the state of Michigan from March 2020 to September 2021. The model was derived from a cohort from the first COVID-19 wave from 1 March to 31 December 2020 (N = 3531) and further validated on a cohort of patients from the second wave from 1 January and 5 September 2021 (N = 2508). We observed a similar incidence of in-hospital VTE in the derivation cohort (7.8%, N = 276) and validation cohort (7.3%, N = 182). Despite the evolution in COVID-19 management over the course of the pandemic, including the use of steroids, Janus kinase inhibitors, interleukin-6 receptor inhibitors, antiviral agents, and anticoagulation treatments, the RAM that we developed shows acceptable discrimination and good calibration. The AUROC of our model is 0.74, which makes the clinical value of our model moderate for the prediction of VTE. However, the predictive performance of our RAM was superior to the SOFA score (AUROC 0.60), which was tested on our cohort for the risk stratification of VTE in COVID-19 patients [15].

The weighted variables in the RAM included presenting heart rate, systolic blood pressure, high and very high D-dimer, low serum albumin, history of PE, and history of DVT, which are readily available parameters for clinicians. One of the most widely used criteria for pulmonary embolism, Wells’ criteria, included tachycardia (HR > 100) [22]; this variable, along with D-dimer, has demonstrated a high predictive value for PE in various studies [23,24] Moreover, systolic blood pressure ≤120 is known to be associated with a worse prognosis in patients with VTE [25]. Hypotension in patients with VTE is likely secondary to vasomotor reflex, causing a decrease in systemic arterial resistance and right ventricular dysfunction, leading to a decreased cardiac output [26]. In addition, hypotension and hypoxemia in the setting of the ventilation–perfusion mismatch and right heart failure can lead to tachycardia in these patients [27,28]. Moreover, the systemic inflammatory cascade seen in SARS-CoV-2 infection can contribute to hypotension, tachycardia, and the risk of VTE [29].

We categorized variables to address the values that were highly skewed, likely due to active COVID-19 viral infection, superimposed bacterial infections, and resulting multiorgan failure (Supplementary Table S2). In patients with suspected PE, D-dimer levels correlate with the probability of PE [30,31]. The meta-analysis by Kollias and colleagues showed that the prevalence of PE in COVID-19 was higher with higher mean D-dimer values (prevalence ratio 1.3 per 1000 ng/mL increase; 95% CI: 1.11–1.50; *p* = 0.002) across the studies [4]. In COVID-19 patients, a D-dimer >3000 ng/L in combination with Wells score >2 is shown to have high specificity in detecting VTE [32]. Here, we observed that two cutoff values for presenting D-dimer, 500 and 2000 ng/mL, had high predictive scores in COVID-19 patients. Serum albumin, which is a known marker of VTE risk, was associated with a higher risk of VTE in our study. Albumin could be associated with higher fibrinogen and factor VIII levels, and shorter activated partial thromboplastin time, therefore reflecting a hypercoagulable state [33]. Moreover, albumin is a known marker of systemic inflammation, which is also seen in COVID-19 infection [7,34]. Albumin is prognostic for hospitalized COVID-19 patients [35], and its administration might have an anticoagulant effect [36], although further studies are needed to explore this. Our results showed that other biomarkers of interest, such as ferritin, interleukin-6, and lactate dehydrogenase, were not significant predictors of VTE. Therefore, physicians may consider reducing routine testing of these markers. We found that a history of VTE, which is known to be associated with severe COVID-19 infection, was a strong predictor of VTE during hospitalization [37].

The efficacy of VTE prophylaxis on clinical outcomes in COVID-19 patients remains inconclusive. Current guidelines support VTE prophylaxis in hospitalized COVID-19 patients [9,10,11,12]; therapeutic anticoagulation is recommended in non-critical patients, whereas prophylactic anticoagulation is recommended in patients with critical illness [12,38]. Moreover, escalation to intermediate- or therapeutic-dose anticoagulation is recommended for deteriorating clinical status, obesity, high thrombotic risk, and when diagnostic imaging is not possible [9,12]. Some retrospective studies found that anticoagulation was associated with decreased mortality [39,40]. The results of a combined multiplatform adaptive randomized control trial by the ATTACC, ACTIV-4a, and REMAP-CAP investigators showed that therapeutic-dose heparin did not improve survival or freedom of cardiovascular or respiratory support than usual-care anticoagulation in critically ill patients [16] but showed survival benefits in non-critically ill patients [17]. The benefit of therapeutic anticoagulation in non-critically ill patients could be explained by the antithrombotic, anti-inflammatory, and potentially antiviral mechanisms of heparin [41,42], whereas, in critically ill patients, heparin was unable to influence the advanced stages of thrombosis, inflammation, and organ damage [43,44,45]. However, other studies have found higher mortality and bleeding in patients receiving intermediate-to-therapeutic-dose anticoagulation, which emphasizes the need for the careful assessment of each patient’s risk profile when prescribing anticoagulation treatment [46,47,48]. In our study, 42% of patients in the derivation and 28.3% of patients in the validation cohort received prophylactic anticoagulation, whereas therapeutic anticoagulation was given in 30.6% of patients in the derivation and 31.5% in the validation cohort. No anticoagulation was given in 27.4% of patients in the derivation and 40.1% of patients in the validation cohort (Supplementary Tables S6 and S7).

We acknowledge that further studies are needed for further external validation in different healthcare settings and countries; however, our model can be useful in scenarios where there is clinical ambiguity on the continuation of anticoagulation for a patient in whom it has already been initiated based on the recent evidence of the beneficial effects of anticoagulation in mild disease and futility with severe disease [16,17]. It can also be particularly helpful for patients who are on high settings of the ventilator or on multiple vasopressors and are not stable enough to undergo a computed tomography angiography exam. Moreover, our prediction model can help in the early risk stratification of patients in settings where early imaging confirmation of VTE is not possible due to isolation precautions or when hospital resources are overwhelmed with a high burden of patients. Furthermore, this RAM can be useful in resource-limited countries where diagnostic testing may not be available. Physicians in such countries could utilize our model to predict VTE based on the commonly tested variables.

Our RAM has both strengths and limitations. Strengths include the large and diverse patient population and the multicenter nature of the study. We collected comprehensive data on baseline demographics, comorbid conditions, social history, vital signs, and laboratory values. Our model consists of variables that are simple, interpretable, and readily available to clinicians on the arrival of patients to the hospital. The variable scores were based on robust statistical computations. Our model can potentially limit the need for the testing of labs, including inflammatory markers, which are often done in clinical practice to determine the risk of VTE. The model can help to identify COVID-19 patients at risk for VTE at the time of admission and thus facilitate better clinical management. It can guide the early initiation of therapeutic anticoagulation for patients identified as at high risk for VTE, especially when a definitive diagnosis cannot be made. We also provided cutoff values for predictors, including D-dimer, albumin, presenting systolic blood pressure, and heart rate. Finally, the RAM showed good performance in the bootstrapped sample and validation cohort, which gave additional strength to our analysis.

The limitations of our study include its retrospective nature, lack of time-to-event analysis, and potential for time-dependent and competing risk bias. To overcome this, we used the first set of parameters or baseline covariates collected at the time of the patient’s admission. Our RAM was built using hospitalized patients; therefore, it lacks generalizability in the outpatient setting. We did not validate our model in independent cohorts; thereby, there is a risk of overestimation of AUROC, and further studies are needed to see our RAM’s performance in other hospitals and countries. Although we compared our RAM with the SOFA score, which was initially validated for DVT in COVID-19 patients [15], we could not compare our model to other VTE models because of the lack of specific data for such comparisons [49,50,51]. Moreover, anticoagulation was not included in our model, as we did not have data on the time-to-event of VTE and the relationship between the timing of VTE and the receipt of anticoagulation in our cohort. The effect of anticoagulation on the risk of VTE and bleeding risk associated with anticoagulation should be explored in future studies. The low incidence of VTE in our study cohort contributed to the low PPV of the RAM; however, the incidence is consistent with other studies [52,53,54]. Lastly, our model is at high risk of type 1 error due to a high false-positive rate, which resulted from low specificity to accommodate for higher sensitivity for the chosen score cutoff in our RAM.

## 5. Conclusions

We derived a novel RAM from a large cohort of patients, using seven important clinical variables, which could be easily applied in clinical practice. This simplified diagnostic approach can help clinicians to risk-stratify COVID-19 patients on admission. It can potentially be used as an adjunct clinical decision support tool for individualizing anticoagulation for high-VTE-risk patient populations. Further studies are needed for the model’s validation in other cohorts and for further direct comparisons of our RAM to other VTE scores in COVID-19 patients.

## Figures and Tables

**Table 1:** Baseline characteristics of all COVID-19 patients.

Variable	All Patients	VTE (N = 276)	No VTE	*p*-Value
	(N = 3531)		(N = 3255)	
**(A)** ** Comparison of patients in derivation cohort stratified by in-hospital venous thromboembolism status.**				
Sex				0.96
Male	1777 (50.3)	138 (50.0)	1639 (50.4)	
Female	1754 (49.7)	138 (50.0)	1616 (49.6)	
Age (years)				0.717
18–39	230 (6.5)	14 (5.1)	216 (6.6)	
40–59	808 (22.9)	65 (23.6)	743 (22.8)	
60–79	1602 (45.4)	123 (44.6)	1479 (45.4)	
≥80	891 (25.2)	74 (26.8)	817 (25.1)	
Race/ethnicity *				0.206
Hispanic	55 (1.6)	1 (0.4)	54 (1.7)	
Other	251 (7.1)	15 (5.4)	236 (7.3)	
White	2034 (57.6)	159 (57.6)	1875 (57.6)	
Black	1191 (33.7)	101 (36.6)	1090 (33.5)	
Body mass index (kg/m^2^)				0.155
<18.5	81 (2.3)	7 (2.5)	74 (2.3)	
18.5–24.9	678 (19.2)	67 (24.3)	611 (18.8)	
25.0–29.9	1060 (30.0)	79 (28.6)	981 (30.1)	
≥30.0	1712 (48.5)	123 (44.6)	1589 (48.8)	
Heart rate				0.001
<100	2173 (61.5)	143 (51.8)	2030 (62.4)	
≥100	1358 (38.5)	133 (48.2)	1225 (37.6)	
Respiratory rate				0.211
<20	1647 (46.6)	124 (44.9)	1523 (46.8)	
20–29	1576 (44.6)	120 (43.5)	1456 (44.7)	
≥30	308 (8.7)	32 (11.6)	276 (8.5)	
Oxygen				0.031
Normal	1516 (42.9)	101 (36.6)	1415 (43.5)	
Low	2015 (57.1)	175 (63.4)	1840 (56.5)	
Systolic blood pressure				0.053
Normal	938 (26.6)	92 (33.3)	846 (26.0)	
Low	100 (2.8)	5 (1.8)	95 (2.9)	
High	1981 (56.1)	142 (51.4)	1839 (56.5)	
Very high	512 (14.5)	37 (13.4)	475 (14.6)	
**Comorbidities**				
Hypertension	2736 (77.5)	213 (77.2)	2523 (77.5)	0.957
Diabetes mellitus	1493 (42.3)	111 (40.2)	1382 (42.5)	0.509
Hyperlipidemia	1685 (47.7)	124 (44.9)	1561 (48.0)	0.366
Coronary artery disease	709 (20.1)	52 (18.8)	657 (20.2)	0.648
Congestive heart failure	454 (12.9)	30 (10.9)	424 (13.0)	0.35
Cerebrovascular accident	455 (12.9)	30 (10.9)	425 (13.1)	0.343
Solid cancer and hematological malignancy	609 (17.2)	61 (22.1)	548 (16.8)	0.032
Autoimmune disease	212 (6.0)	18 (6.5)	194 (6.0)	0.806
Liver disease *	29 (0.8)	5 (1.8)	24 (0.7)	0.071
Interstitial lung disease *	19 (0.5)	1 (0.4)	18 (0.6)	1
Chronic obstructive pulmonary disease	509 (14.4)	30 (10.9)	479 (14.7)	0.097
Atrial fibrillation	371 (10.5)	22 (8.0)	349 (10.7)	0.184
Deep vein thrombosis	251 (7.1)	45 (16.3)	206 (6.3)	<0.001
Pulmonary embolism	164 (4.6)	34 (12.3)	130 (4.0)	<0.001
Thyroid disease	509 (14.4)	44 (15.9)	465 (14.3)	0.507
**Social history**				
Smoker	272 (7.7)	20 (7.2)	252 (7.7)	0.858
Alcohol use	61 (1.7)	7 (2.5)	54 (1.7)	0.329
**Presenting laboratory values**				
Leukocytes				<0.001
Normal	2666 (75.5)	182 (65.9)	2484 (76.3)	
Low	314 (8.9)	22 (8.0)	292 (9.0)	
High	551 (15.6)	72 (26.1)	479 (14.7)	
Lymphocytes *				0.163
Normal	1377 (39.0)	116 (42.0)	1261 (38.7)	
Low	2110 (59.8)	154 (55.8)	1956 (60.1)	
High	44 (1.3)	6 (2.2)	38 (1.2)	
Neutrophils				<0.001
Normal	3026 (85.7)	211 (76.4)	2815 (86.5)	
Low	77 (2.2)	8 (2.9)	69 (2.1)	
High	428 (12.1)	57 (20.7)	371 (11.4)	
Neutrophil/Lymphocyte ratio *				0.027
Normal	877 (24.8)	58 (21.0)	819 (25.2)	
Low	34 (1.0)	3 (1.1)	31 (1.0)	
Mild	1564 (44.3)	111 (40.2)	1453 (44.6)	
Moderate	729 (20.7)	66 (23.9)	663 (20.4)	
Severe	327 (9.3)	38 (13.8)	289 (8.9)	
B-type natriuretic peptide				0.006
Normal	2221 (62.9)	152 (55.1)	2069 (63.6)	
High	1310 (37.1)	124 (44.9)	1186 (36.4)	
C-reactive protein				0.011
Normal	86 (2.4)	0 (0.0)	86 (2.6)	
High	3445 (97.6)	276 (100.0)	3169 (97.4)	
D-dimer				<0.001
Normal	1458 (41.3)	40 (14.5)	1418 (43.6)	
High	1707 (48.3)	152 (55.1)	1555 (47.8)	
Very high	366 (10.4)	84 (30.4)	282 (8.7)	
Ferritin				0.63
Normal	1475 (41.8)	111 (40.2)	1364 (41.9)	
High	2056 (58.2)	165 (59.8)	1891 (58.1)	
Lactate dehydrogenase				0.001
Normal	703 (19.9)	38 (13.8)	665 (20.4)	
High	2828 (80.1)	238 (86.2)	2590 (79.6)	
Blood urea nitrogen				0.04
Normal	2105 (59.6)	148 (53.6)	1957 (60.1)	
High	1426 (40.4)	128 (46.4)	1298 (39.9)	
Creatinine				0.375
Normal	2494 (70.6)	188 (68.1)	2306 (70.8)	
High	1037 (29.4)	88 (31.9)	949 (29.2)	
Total bilirubin				0.108
Normal	3364 (95.3)	257 (93.1)	3107 (95.5)	
High	167 (4.7)	19 (6.9)	148 (4.5)	
Aspartate transaminase				0.616
Normal	1545 (43.8)	113 (40.9)	1432 (44.0)	
Low	71 (2.0)	6 (2.2)	65 (2.0)	
High	1915 (54.2)	157 (56.9)	1758 (54.0)	
Alanine transaminase				0.065
Normal	2346 (66.4)	167 (60.5)	2179 (66.9)	
Low	310 (8.8)	32 (11.6)	278 (8.5)	
High	875 (24.8)	77 (27.9)	798 (24.5)	
Alkaline phosphatase				0.437
Normal	3108 (88.0)	240 (87.0)	2868 (88.1)	
Low	178 (5.0)	12 (4.3)	166 (5.1)	
High	245 (7.0)	24 (8.7)	221 (6.8)	
Albumin				<0.001
Good	916 (25.9)	43 (15.6)	873 (26.8)	
Low	371 (10.5)	54 (19.6)	317 (9.7)	
Borderline	2244 (63.6)	179 (64.9)	2065 (63.4)	
Troponin-I				0.129
Normal	2103 (59.6)	152 (55.1)	1951 (59.9)	
High	1428 (40.4)	124 (44.9)	1304 (40.1)	
Creatine phosphokinase				0.889
Normal	2347 (66.5)	185 (67.0)	2162 (66.4)	
High	1184 (33.5)	91 (33.0)	1093 (33.6)	
Interleukin-6				0.031
Normal	326 (9.2)	15 (5.4)	311 (9.6)	
High	3205 (90.8)	261 (94.6)	2944 (90.4)	
Lactate *				0.342
Normal	2755 (78.0)	214 (77.5)	2541 (78.1)	
Low	4 (0.1)	1 (0.4)	3 (0.1)	
High	772 (21.9)	61 (22.1)	711 (21.8)	
Procalcitonin				0.602
Normal	2296 (65.0)	175 (63.4)	2121 (65.2)	
High	1235 (35.0)	101 (36.6)	1134 (34.8)	
Potassium				0.028
Normal	2644 (74.9)	197 (71.4)	2447 (75.2)	
Hypokalemia	652 (18.5)	50 (18.1)	602 (18.5)	
Hyperkalemia	235 (6.7)	29 (10.5)	206 (6.3)	
Platelet Count				<0.001
Normal	2793 (79.1)	212 (76.8)	2581 (79.3)	
Low	610 (17.3)	41 (14.9)	569 (17.5)	
High	128 (3.6)	23 (8.3)	105 (3.2)	
**(B) Comparison of patients in validation cohort stratified by in-hospital venous thromboembolism status.**				
Sex				0.604
Male	1187 (47.3)	90 (49.5)	1097 (47.2)	
Female	1321 (52.7)	92 (50.5)	1229 (52.8)	
Age (years)				0.162
18–39	369 (14.7)	18 (9.9)	351 (15.1)	
40–59	734 (29.3)	51 (28.0)	683 (29.4)	
60–79	989 (39.4)	76 (41.8)	913 (39.3)	
≥80	416 (16.6)	37 (20.3)	379 (16.3)	
Race/ethnicity *				0.324
Hispanic	63 (2.5)	4 (2.2)	59 (2.5)	
Other	182 (7.3)	15 (8.2)	167 (7.2)	
White	1475 (58.8)	96 (52.7)	1379 (59.3)	
Black	788 (31.4)	67 (36.8)	721 (31.0)	
Body mass index (kg/m^2^)				0.422
<18.5	72 (2.9)	8 (4.4)	64 (2.8)	
18.5–24.9	448 (17.9)	28 (15.4)	420 (18.1)	
25.0–29.9	708 (28.2)	56 (30.8)	652 (28.0)	
≥30.0	1280 (51.0)	90 (49.5)	1190 (51.2)	
Heart rate				0.034
<100	1542 (61.5)	98 (53.8)	1444 (62.1)	
≥100	966 (38.5)	84 (46.2)	882 (37.9)	
Respiratory rate				<0.001
<20	1385 (55.2)	75 (41.2)	1310 (56.3)	
20–29	1004 (40.0)	84 (46.2)	920 (39.6)	
≥30	119 (4.7)	23 (12.6)	96 (4.1)	
Oxygen saturation				0.001
Normal	1216 (48.5)	66 (36.3)	1150 (49.4)	
Low	1292 (51.5)	116 (63.7)	1176 (50.6)	
Systolic blood pressure *				0.891
Normal	690 (27.5)	49 (26.9)	641 (27.6)	
Low	55 (2.2)	5 (2.7)	50 (2.1)	
High	1422 (56.7)	105 (57.7)	1317 (56.6)	
Very high	341 (13.6)	23 (12.6)	318 (13.7)	
**Comorbidities**				
Hypertension	1727 (68.9)	134 (73.6)	1593 (68.5)	0.174
Diabetes mellitus	991 (39.5)	70 (38.5)	921 (39.6)	0.824
Hyperlipidemia	1069 (42.6)	73 (40.1)	996 (42.8)	0.526
Coronary artery disease	386 (15.4)	27 (14.8)	359 (15.4)	0.913
Congestive heart failure	352 (14.0)	30 (16.5)	322 (13.8)	0.381
Cerebrovascular accident	318 (12.7)	23 (12.6)	295 (12.7)	1
Solid cancer and hematological malignancy	359 (14.3)	33 (18.1)	326 (14.0)	0.156
Autoimmune disease	122 (4.9)	5 (2.7)	117 (5.0)	0.23
Liver disease *	42 (1.7)	4 (2.2)	38 (1.6)	0.542
Interstitial lung disease *	12 (0.5)	0 (0.0)	12 (0.5)	1
Chronic obstructive pulmonary disease	307 (12.2)	23 (12.6)	284 (12.2)	0.958
Atrial fibrillation	318 (12.7)	24 (13.2)	294 (12.6)	0.922
Deep vein thrombosis	256 (10.2)	28 (15.4)	228 (9.8)	0.023
Pulmonary embolism	185 (7.4)	37 (20.3)	148 (6.4)	<0.001
Thyroid disease	352 (14.0)	24 (13.2)	328 (14.1)	0.817
Social history				
Smoker	265 (10.6)	12 (6.6)	253 (10.9)	0.092
Alcohol use	88 (3.5)	4 (2.2)	84 (3.6)	0.43
**Presenting laboratory values**				
Leukocytes				<0.001
Normal	1899 (75.7)	112 (61.5)	1787 (76.8)	
Low	252 (10.0)	15 (8.2)	237 (10.2)	
High	357 (14.2)	55 (30.2)	302 (13.0)	
Lymphocytes				<0.001
Normal	1405 (56.0)	80 (44.0)	1325 (57.0)	
Low	732 (29.2)	56 (30.8)	676 (29.1)	
High	371 (14.8)	46 (25.3)	325 (14.0)	
Neutrophils *				<0.001
Normal	2016 (80.4)	125 (68.7)	1891 (81.3)	
Low	194 (7.7)	15 (8.2)	179 (7.7)	
High	298 (11.9)	42 (23.1)	256 (11.0)	
Neutrophil/Lymphocyte ratio *				0.029
Normal	1024 (40.8)	60 (33.0)	964 (41.4)	
Low	330 (13.2)	30 (16.5)	300 (12.9)	
Mild	7883 (31.2)	55 (30.2)	728 (31.3)	
Moderate	254 (10.1)	22 (12.1)	232 (10.0)	
Severe	117 (4.7)	15 (8.2)	102 (4.4)	
B-type natriuretic peptide				0.048
Normal	1341 (53.5)	84 (46.2)	1257 (54.0)	
High	1167 (46.5)	98 (53.8)	1069 (46.0)	
C-reactive protein				0.415
Normal	105 (4.2)	5 (2.7)	100 (4.3)	
High	2403 (95.8)	177 (97.3)	2226 (95.7)	
D-dimer				<0.001
Normal	1276 (50.9)	34 (18.7)	1242 (53.4)	
High	1058 (42.2)	99 (54.4)	959 (41.2)	
Very high	174 (6.9)	49 (26.9)	125 (5.4)	
Ferritin				0.101
Normal	1187 (47.3)	75 (41.2)	1112 (47.8)	
High	1321 (52.7)	107 (58.8)	1214 (52.2)	
Lactate dehydrogenase				0.002
Normal	452 (18.0)	17 (9.3)	435 (18.7)	
High	2056 (82.0)	165 (90.7)	1891 (81.3)	
Blood urea nitrogen				<0.001
Normal	1685 (67.2)	99 (54.4)	1586 (68.2)	
High	823 (32.8)	83 (45.6)	740 (31.8)	
Creatinine				0.026
Normal	1938 (77.3)	128 (70.3)	1810 (77.8)	
High	570 (22.7)	54 (29.7)	516 (22.2)	
Total bilirubin				0.138
Normal	2369 (94.5)	167 (91.8)	2202 (94.7)	
High	139 (5.5)	15 (8.2)	124 (5.3)	
Aspartate transaminase				0.084
Normal	1410 (56.2)	88 (48.4)	1322 (56.8)	
Low	73 (2.9)	6 (3.3)	67 (2.9)	
High	1025 (40.9)	88 (48.4)	937 (40.3)	
Alanine transaminase				0.732
Normal	2012 (80.2)	148 (81.3)	1864 (80.1)	
Low	143 (5.7)	8 (4.4)	135 (5.8)	
High	353 (14.1)	26 (14.3)	327 (14.1)	
Alkaline phosphatase				0.793
Normal	2182 (87.0)	156 (85.7)	2026 (87.1)	
Low	112 (4.5)	8 (4.4)	104 (4.5)	
High	214 (8.5)	18 (9.9)	196 (8.4)	
Albumin				<.001
Good	748 (29.8)	32 (17.6)	716 (30.8)	
Low	254 (10.1)	30 (16.5)	224 (9.6)	
Borderline	1506 (60.0)	120 (65.9)	1386 (59.6)	
Troponin-I				0.001
Normal	645 (25.7)	27 (14.8)	618 (26.6)	
High	1863 (74.3)	155 (85.2)	1708 (73.4)	
Creatine phosphokinase				0.764
Normal	1658 (66.1)	118 (64.8)	1540 (66.2)	
High	850 (33.9)	64 (35.2)	786 (33.8)	
Interleukin 6				0.089
Normal	165 (6.6)	6 (3.3)	159 (6.8)	
High	2343 (93.4)	176 (96.7)	2167 (93.2)	
Lactate *				0.636
Normal	1842 (73.4)	129 (70.9)	1713 (73.6)	
Low	7 (0.3)	0 (0.0)	7 (0.3)	
High	659 (26.3)	53 (29.1)	606 (26.1)	
Procalcitonin				0.271
Normal	1400 (55.8)	94 (51.6)	1306 (56.1)	
High	1108 (44.2)	88 (48.4)	1020 (43.9)	
Potassium				0.663
Normal	1900 (75.8)	133 (73.1)	1767 (76.0)	
Hypokalemia	479 (19.1)	38 (20.9)	441 (19.0)	
Hyperkalemia	129 (5.1)	11 (6.0)	118 (5.1)	
Platelet Count				0.247
Normal	2008 (80.1)	145 (79.7)	1863 (80.1)	
Low	425 (16.9)	28 (15.4)	397 (17.1)	
High	75 (3.0)	9 (4.9)	66 (2.8)	

* Fisher exact tests were used for these variables; chi-square tests were used otherwise. Abbreviation: VTE, venous thromboembolism.

**Table 2:** The 3D-PAST risk assessment model with assigned scores.

Risk Assessment Model		
**Variables**	**Values**	**Point**
		**(Total Score: 14)**
**D**eep vein thrombosis history	Yes	+2
High **D**-dimer on admission	>500–2000 ng/mL	+2
Very high **D**-dimer on admission	>2000 ng/mL	+5
**P**ulmonary embolism history	Yes	+2
Low **A**lbumin	<3.5 g/dL	+1
**S**ystolic blood pressure	<120 mmHg	+1
**T**achycardia	>100 bpm	+1

3D-PAST risk assessment mode score is delineated as DVT **H**istory = 2; High **D**-Dimer (>500–2000 ng/mL) = 2; Very **H**igh D-Dimer (>2000 ng/mL) = 5; **P**E History = 2; Low **A**lbumin (<3.5 g/dL) = 1; **S**ystolic Blood Pressure <120 mmHg = 1; **T**achycardia (heart rate >100 bpm) = 1.

## Data Availability

Data are available from the authors on request.

## Supplementary Materials

The following supporting information can be downloaded at: https://www.mdpi.com/article/10.3390/jcm11143949/s1, Figure S1: Heat map showing percentages of missing data in the study cohort. Figure S2: (A) Area under the receiver operating characteristic of derivation cohort Abbreviations: AUC, area under the curve; (B) Area under the receiver operating characteristic of boot-strapped sample, (C) Area under the receiver operating characteristic of validation cohort. Figure S3: Area under the receiver operating characteristic of SOFA score validated for risk stratification of acute venous thromboembolism in our study cohort. Table S1: International Classification of Diseases–Tenth Revision Codes and other identification methods used for identification of patients. Table S2: Categorization of the variables used in model building. Table S3: Baseline characteristics of bootstrapped sample. Table S4: Variables selected by LASSO. Table S5: The sensitivity and specificity of different cut-off scores in the model. Table S6: Anticoagulation treatments received by patients in the validation cohort. Table S7: Anticoagulation treatments received by patients in the validation cohort.

## Abbreviations

AIC	Akaike information criterion
AUROC	Area under the receiver operating characteristic
CI	Confidence interval
DVT	Deep vein thrombosis
ICD-10	International Classification of Diseases—Tenth Revision
LASSO	Least Absolute Shrinkage and Selection Operator
MICE	Multivariate imputation by chained equations
NPV	Negative predictive value
PCR	Polymerase chain reaction
PE	Pulmonary embolism
PPV	Positive predictive value
RAM	Risk assessment model
SOFA	Sequential Organ Failure Assessment
SMCRD	Southeast Michigan COVID-19 Consortium Registry Database
VTE	Venous thromboembolism
