# Supplementary material for: 3D-PAST: Risk Assessment Model for Predicting Venous Thromboembolism in COVID-19
Source: J Clin Med. 2022 Jul 7;11(14):3949. doi: 10.3390/jcm11143949 (PMC9325096; doi:10.3390/jcm11143949)
Supplement: Supplementary file 1 [file jcm-11-03949-s001.zip › jcm-1672526-supplementary.pdf]

SUPPLEMENTARY MATERIALS

3D-PAST: Risk Assessment Model for Predicting Venous Thromboembolism in COVID-19

Yi Lee, Qasim Jehangir, Chun-Hui Lin, Pin Li, Anupam A. Sule, Laila Poisson, Venugopal Balijepally, Abdul R. Halabi, Kiritkumar Patel, Geetha Krishnamoorthy, Girish B. Nair

Table of Contents

Figure S1: Heat map showing percentages of missing data in the study cohort

Figure S2: (A) Area under the receiver operating characteristic of derivation cohort Abbreviations: AUC, area under the curve; (B) Area under the receiver operating characteristic of boot-strapped sample, (C) Area under the receiver operating characteristic of validation cohort

Figure S3: Area under the receiver operating characteristic of SOFA score validated for risk stratification of acute venous thromboembolism in our study cohort

Table S1: International Classification of Diseases–Tenth Revision Codes and other identification methods used for identification of patients

Table S2: Categorization of the variables used in model building

Table S3: Baseline characteristics of bootstrapped sample

Table S4: Variables selected by LASSO

Table S5: The sensitivity and specificity of different cut-off scores in the model

Table S6: Anticoagulation treatments received by patients in the validation cohort

Table S7: Anticoagulation treatments received by patients in the validation cohort

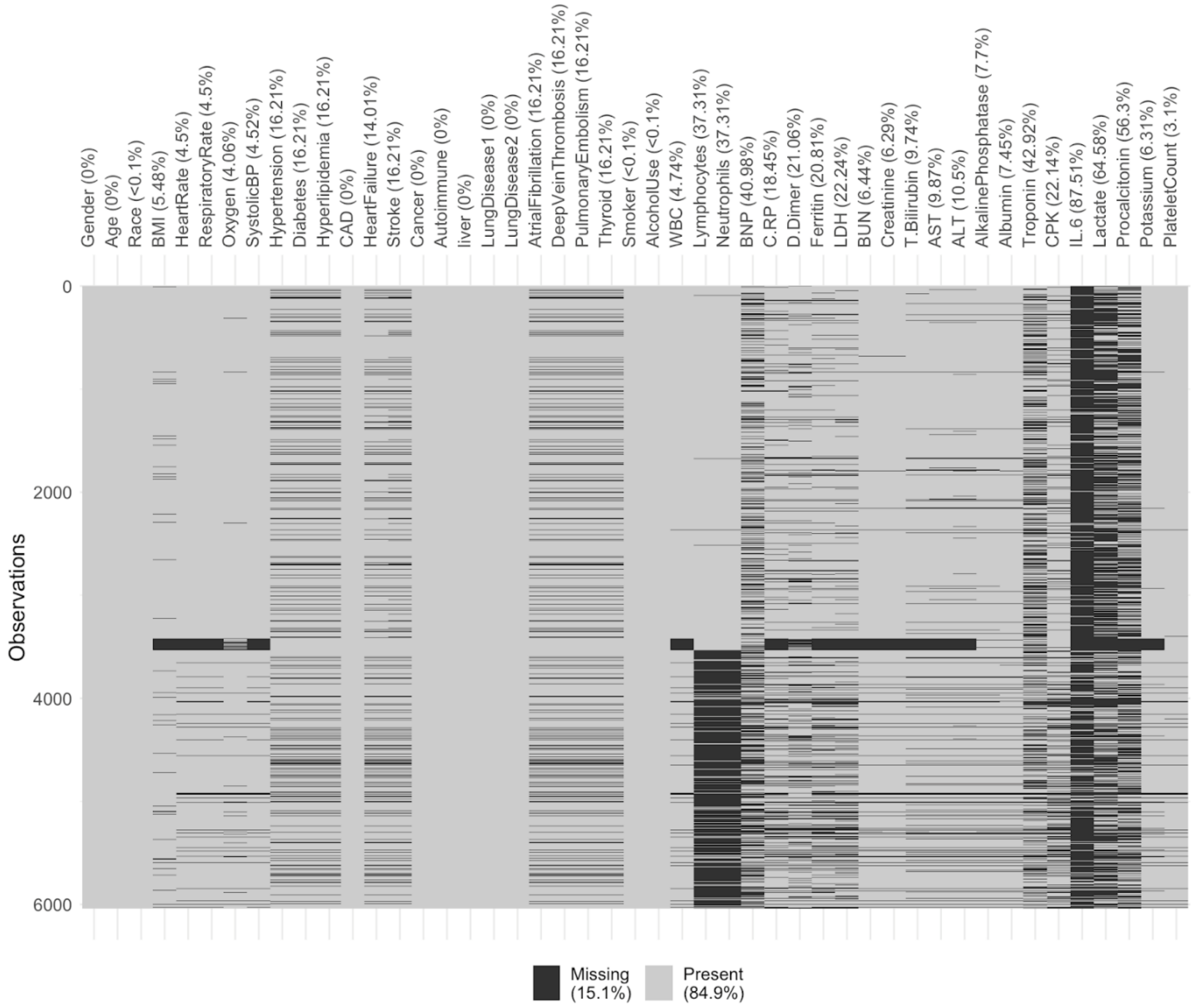

Figure S1. Heat map showing percentages of missing data in the study cohort. The percentages of missingness in each column are shown. There was 15.1% missingness in the whole data.

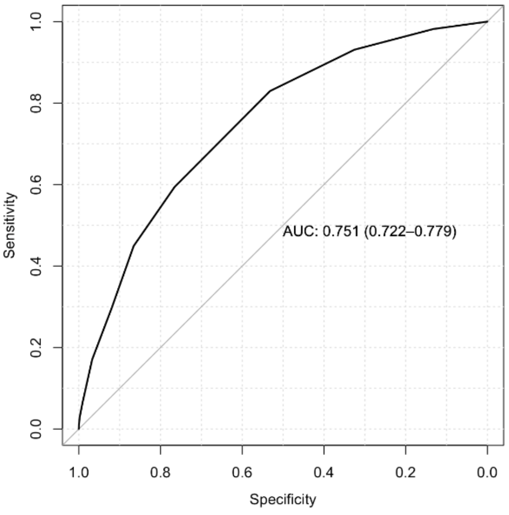

(a)

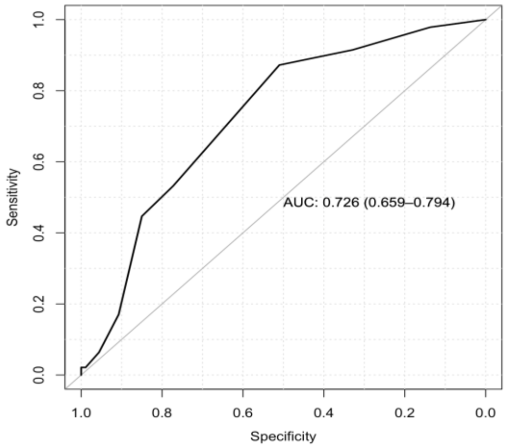

(b)

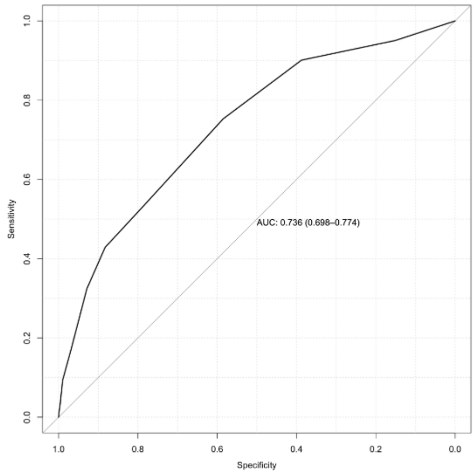

(c)

Figure S2. A. Area under the receiver operating characteristic of derivation cohort Abbreviations: AUC, area under the curve; **B**. Area under the receiver operating characteristic of

bootstrapped sample, **C**. Area under the receiver operating characteristic of validation cohort.

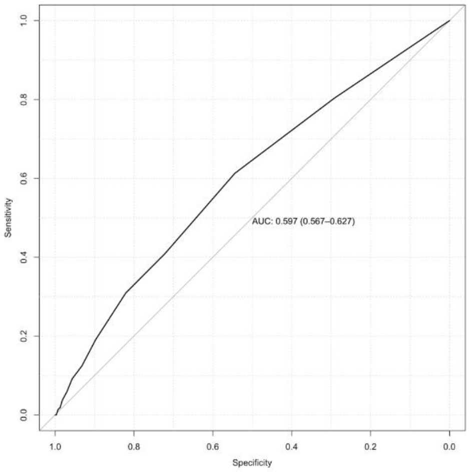

Figure S3 - Area under the receiver operating characteristic of SOFA score validated for risk stratification of acute venous thromboembolism in our study cohort.

Table S1. International Classification of Diseases–Tenth Revision Codes and other identification methods used for identification of patients.

Variable	ICD-10 & consortium standard-text codes
Acute pulmonary embolism	Discharge Diagnosis: Acute pulmonary embolism<br><br>I26
Acute deep vein thrombosis	Discharge Diagnosis: Deep vein thrombosis<br><br>I82.0 I82.2 I82.21 I82.210 I82.22 I82.220 I82.29 I82.29 I82.290 I82.3 I82.4 I82.6 I82.60 I82.62 I82.A I82.A1 I82.B I82.B1 I82.C I82.C1 I82.8 I82.89 I82.890 I82.9 I82.90

Table S2. Categorization of the variables used in model building.

Variables	*Categorical or Continuous*	*Definition*
Age (years)	Categories (Ref: 19-39)	19-39
		40-59
		60-79
		≥80
Body Mass Index (kg/m²)	Categories (Ref: 18.5-24.9)	<18.5
		18.5-24.9
		25.0-29.9
		≥30.0
Sex	Categories (Ref: Male)	Male
		Female
Race	Categories (Ref: Black)	Black
		White
		Hispanic
		Other
Vital Signs		
Heart Rate (beats/min)	Categories (Ref: <100)	<100: 0
		≥100: 1
Respiratory Rate (breaths/min)	Categories (Ref: <20)	<20
		20-29
		≥30
Oxygen Saturation	Categories (Ref: Normal)	Normal: O2 Saturation >94% And No Oxygen Device
		Low: O2 Saturation ≤94 And Any Oxygen Device Use
Systolic Blood Pressure (mmHg)	Categories (Ref: Normal)	Low: <90
		Normal: 90-119
		High: 120-159
		Very High: ≥160
Comorbidities	Categories (Ref: No)	
Hypertension	Y/N	
Diabetes Mellitus	Y/N	
Hyperlipidemia	Y/N	
Coronary Artery Disease	Y/N	
Congestive Heart Failure	Y/N	
Cerebrovascular Accident	Y/N	Stroke And Transient Ischemic Attack
Cancer And Hematological Malignancies	Y/N	Includes Solid Tumor and Hematologic Malignancies I.E., Leukemia, Lymphoma

Autoimmune Disease	Y/N	Rheumatoid Arthritis, Psoriatic Arthritis, Spondylarthritis, Systemic Lupus Erythematosus, Sjögren's Syndrome, Scleroderma, Polymyalgia Rheumatica, Mixed Connective Tissue Disease, Dermatomyositis, Polymyositis, Polyarteritis Nodosa, Vasculitis, Lupus, Autoimmune Hepatitis, Other Autoimmune Disease
Liver Disease	Y/N	Cirrhosis, Non-Alcoholic Steatohepatitis, Ascites
Interstitial Lung Disease	Y/N	Pulmonary Fibrosis
Chronic Obstructive Pulmonary Disease	Y/N	Chronic Bronchitis, Emphysema
Atrial Fibrillation	Y/N	
Deep Vein Thrombosis	Y/N	
Pulmonary Embolism	Y/N	
Thyroid Disease	Y/N	Hypothyroidism, Hyperthyroidism
Social History		
History Of Smoking	Y/N	
History Of Alcohol Use	Y/N	
Labs On Presentation		
Leukocyte (K/uL)	Categories (Ref: Normal)	Low: <3.7
		Normal: ≥3.7 And ≤11
		High: >11
Lymphocytes (K/uL)	Categories (Ref: Normal)	Low: <1
		Normal: 1.0-3.5
		High: >3.5
Neutrophils (K/uL)	Categories (Ref: Normal)	Low: <1.5
		Normal: 1.5 - 10
		High: >10
Neutrophil-Lymphocyte Ratio	Categories (Ref: Normal)	Low: <0.8
		Normal: 0.8-3.49
		Mild: 3.5-8.99
		Moderate: 9-17.99
		Severe: >18
B-Type Natriuretic Peptide (pg/mL)	Categories (Ref: Normal)	Normal: 0-100
		High: >100
C-Reactive Protein (mg/dL)	Categories (Ref: Normal)	Normal: 0-0.5
		High: >0.5
D-Dimer (ng/mL)	Categories (Ref: Normal)	Normal: 0-500
		High: >500-2000

		Very High: >2000
Ferritin (ng/mL)	Categories (Ref: Normal)	Normal: ≤336
		High: >336
Lactate Dehydrogenase (U/L)	Categories (Ref: Normal)	Normal ≤192
		High: >192
Blood Urea Nitrogen (mg/dL)	Categories (Ref: Normal)	Normal: ≤23
		High: >23
Creatinine (mg/dL)	Categories (Ref: Normal)	Normal: ≤1.40
		High: >1.40
Total Bilirubin (mg/dL)	Categories (Ref: Normal)	Normal: 0.0-1.6
		High: >1.6
Aspartate Aminotransferase (U/L)	Categories (Ref: Normal)	Low: <15
		Normal: 15-41
		High: >41
Alanine Aminotransferase (U/L)	Categories (Ref: Normal)	Low: <10
		Normal: 10-63
		High: >63
Alkaline Phosphatase (U/L)	Categories (Ref: Normal)	Low: <38
		Normal: 38-126
		High: >126
Albumin (mg/dL)	Categories (Ref: Normal)	Low: <3.5
		Normal: ≥3.5
Troponin-I (ng/mL)	Categories (Ref: Normal)	Normal: 0-0.03 NG/ML
		High: 0.04-0.09 NG/ML
		Higher: >0.09 NG/ML
Creatine Phosphokinase (U/L)	Categories (Ref: Normal)	Normal: ≤280 In Males Or ≤155 In Females
		High: >280 In Males Or >155 In Females
Interleukin 6 (pg/mL)	Categories (Ref: Normal)	Normal: 0-5
		High: >5
Lactate (mmol/L)	Categories (Ref: Normal)	Normal: ≤2.2
		High: >2.2
Procalcitonin (ng/mL)	Categories (Ref: Normal)	Normal: 0-0.5
		High: >0.5
Potassium (meq/L)	Categories (Ref: Normal)	Hypokalemia: <3.5
		Normal: 3.5-5
		Hyperkalemia: >5
Platelet Count (K/UL)	Categories (Ref: Normal)	Low: <140
		Normal: 140-440
		High: >440

Table S3. Comparison of patients in bootstrapped sample stratified by in-hospital venous thromboembolism status.

Variable	All patients (N = 500)	Venous thromboembolism (N = 47)	No venous thromboembolism (N = 453)	p-value
Sex				0.101
Male	243 (48.6)	17 (36.2)	226 (49.9)	
Female	257 (51.4)	30 (63.8)	227 (50.1)	
Age (years)*				0.195
18-39	36 (7.2)	2 (4.3)	34 (7.5)	
40-59	123 (24.6)	14 (29.8)	109 (24.1)	
60-79	219 (43.8)	15 (31.9)	204 (45.0)	
>=80	122 (24.4)	16 (34.0)	106 (23.4)	
Race/ethnicity*				0.329
Hispanic	10 (2.0)	0 (0.0)	10 (2.2)	
Other	43 (8.6)	2 (4.3)	41 (9.1)	
White	298 (59.6)	34 (72.3)	264 (58.3)	
Black	149 (29.8)	11 (23.4)	138 (30.5)	
Body mass index (kg/m^2)*				0.274
<18.5	10 (2.0)	0 (0.0)	10 (2.2)	
18.5-24.9	85 (17.0)	11 (23.4)	74 (16.3)	
25.0-29.9	155 (31.0)	10 (21.3)	145 (32.0)	
>=30.0	250 (50.0)	26 (55.3)	224 (49.4)	
Heart rate				0.873
<100	298 (59.6)	27 (57.4)	271 (59.8)	
>=100	202 (40.4)	20 (42.6)	182 (40.2)	
Respiratory rate*				0.973
<20	221 (44.2)	20 (42.6)	201 (44.4)	
20-29	233 (46.6)	23 (48.9)	210 (46.4)	
>=30	46 (9.2)	4 (8.5)	42 (9.3)	
Oxygen saturation				0.035
Normal	216 (43.2)	13 (27.7)	203 (44.8)	
Low	284 (56.8)	34 (72.3)	250 (55.2)	
Systolic blood pressure*				0.29
Normal	131 (26.2)	17 (36.2)	114 (25.2)	
Low	14 (2.8)	1 (2.1)	13 (2.9)	
High	282 (564)	21 (44.7)	261 (57.6)	
Very high	73 (14.6)	8 (17.0)	65 (14.3)	
**Comorbidities**				
Hypertension	386 (77.2)	38 (80.9)	348 (76.8)	0.657

Diabetes mellitus	214 (42.8)	20 (42.6)	194 (42.8)	1
Hyperlipidemia	238 (47.6)	24 (51.1)	214 (47.2)	0.729
Coronary artery disease	90 (18.0)	10 (21.3)	80 (17.7)	0.678
Congestive heart failure	64 (12.8)	5 (10.6)	59 (13.0)	0.813
Cerebrovascular accident	55 (11.0)	7 (14.9)	48 (10.6)	0.515
Solid cancer and hematological malignancy	93 (18.6)	9 (19.1)	84 (18.5)	1
Autoimmune disease*	26 (5.2)	3 (6.4)	23 (5.1)	0.726
Liver disease*	5 (1.0)	0 (0.0)	5 (1.1)	1
Interstitial lung disease*	4 (00.8)	0 (0.0)	4 (0.9)	1
Chronic obstructive pulmonary disease	81 (16.2)	3 (6.4)	78 (17.2)	0.087
Atrial fibrillation*	49 (9.8)	3 (6.4)	46 (10.2)	0.605
Deep vein thrombosis*	29 (5.8)	6 (12.8)	23 (5.1)	0.044
Pulmonary embolism*	20 (4.0)	2 (4.3)	18 (4.0)	1
Thyroid disease	82 (16.4)	6 (12.8)	76 (16.8)	0.617
Social history				
Smoker*	38 (7.6)	4 (8.5)	34 (7.5)	0.772
Alcohol use*	11 (2.2)	1 (2.1)	10 (2.2)	1
**Presenting laboratory values**				
Leukocytes*				0.001
Normal	376 (75.2)	27 (57.4)	349 (77.0)	
Low	43 (8.6)	3 (6.4)	40 (8.8)	
High	81 (16.2)	17 (36.2)	64 (14.1)	
Lymphocytes*				0.124
Normal	227 (45.4)	28 (59.6)	199 (43.9)	
Low	266 (53.2)	19 (40.4)	247 (54.5)	
High	7 (1.4)	0 (0.0)	7 (1.5)	
Neutrophils*				<0.001
Normal	424 (84.8)	30 (63.8)	394 (87.0)	
Low	13 (2.6)	2 (4.3)	11 (2.4)	
High	63 (12.6)	15 (31.9)	48 (10.6)	
Neutrophil/Lymphocyte ratio*				0.385
Normal	149 (29.8)	10 (21.3)	139 (30.7)	
Low	7 (1.4)	0 (0.0)	7 (1.5)	
Mild	203 (40.6)	24 (51.1)	179 (39.5)	
Moderate	104 (20.8)	8 (17.0)	96 (21.2)	

Severe	37 (7.4)	5 (10.6)	32 (7.1)	
B-type natriuretic peptide				0.09
Normal	337 (67.4)	26 (55.3)	311 (68.7)	
High	163 (32.6)	21 (44.7)	142 (31.3)	
C-reactive protein*				0.622
Normal	13 (2.6)	0 (0.0)	13 (2.9)	
High	487 (97.4)	47 (100.0)	440 (97.1)	
D-dimer				<0.001
Normal	199 (39.8)	5 (10.6)	194 (42.8)	
High	242 (48.4)	30 (63.8)	212 (46.8)	
Very High	59 (11.8)	12 (25.5)	47 (10.4)	
Ferritin				0.345
Normal	228 (45.6)	25 (53.2)	203 (44.8)	
High	272 (54.4)	22 (46.8)	250 (55.2)	
Lactate dehydrogenase				0.588
Normal	117 (23.4)	9 (19.1)	108 (23.8)	
High	383 (76.6)	38 (80.9)	345 (76.2)	
Blood urea nitrogen				0.021
Normal	297 (59.4)	20 (42.6)	277 (61.1)	
High	203 (40.6)	27 (57.4)	176 (38.9)	
Creatinine				0.254
Normal	360 (72.0)	30 (63.8)	330 (72.8)	
High	140 (28.0)	17 (36.2)	123 (27.2)	
Total bilirubin*				0.451
Normal	478 (95.6)	44 (93.6)	434 (95.8)	
High	22 (4.4)	3 (6.4)	19 (4.2)	
Aspartate transaminase*				0.57
Normal	242 (48.4)	26 (55.3)	216 (47.7)	
Low	4 (0.8)	0 (0.0)	4 (0.9)	
High	254 (50.8)	21 (44.7)	233 (51.4)	
Alanine transaminase *				0.006
Normal	336 (67.2)	24 (51.1)	312 (68.9)	
Low	50 (10.0)	11 (23.4)	39 (8.6)	
High	114 (22.8)	12 (25.5)	102 (22.5)	
Alkaline phosphatase*				0.566
Normal	425 (85.0)	43 (91.5)	382 (84.3)	
Low	26 (5.2)	1 (2.1)	25 (5.5)	
High	49 (9.8)	3 (6.4)	46 (10.2)	

Albumin*				0.13
Good	136 (27.2)	8 (17.0)	128 (28.3)	
Low	47 (9.4)	7 (14.9)	40 (8.8)	
Borderline	317 (63.4)	32 (68.1)	285 (62.9)	
Troponin-I				0.212
Normal	303 (60.6)	24 (51.1)	279 (61.6)	
High	197 (39.4)	23 (48.9)	174 (38.4)	
Creatine phosphokinase				0.343
Normal	324 (64.8)	27 (57.4)	297 (65.6)	
High	176 (35.2)	20 (42.6)	156 (34.4)	
Interleukin 6*				0.024
Normal	41 (8.2)	0 (0.0)	41 (9.1)	
High	459 (91.8)	47 (100.0)	412 (90.9)	
Lactate				0.223
Normal	372(74.4)	31 (66.0)	341 (75.3)	
Low	0 (0)	0 (0.0)	0 (0.0)	
High	128 (25.6)	16 (34.0)	112 (24.7)	
Procalcitonin				0.893
Normal	320 (64.0)	31 (66.0)	289 (63.8)	
High	180 (36.0)	16 (34.0)	164 (36.2)	
Potassium*				0.025
Normal	382 (76.4)	31 (66.0)	351 (77.5)	
Hypokalemia	83 (16.6)	8 (17.0)	75 (16.6)	
Hyperkalemia	35 (7.0)	8 (17.0)	27 (6.0)	
Platelet count*				<0.001
Normal	409 (81.8)	36 (76.6)	373 (82.3)	
Low	77 (15.4)	4 (8.5)	73 (16.1)	
High	14 (2.8)	7 (14.9)	7 (1.5)	

*Fisher-exact tests were used for these variables; chi-square tests were used otherwise.

Table S4. Variables selected by LASSO.

Age	History of pulmonary embolism	Alanine transaminase
Body mass index	Thyroid disease	Alkaline phosphatase
Heart rate	Leukocytes	Albumin
Oxygen saturation	Lymphocyte count	Troponin-I
Systolic blood pressure	Neutrophil count	Creatinine phosphokinase
Hyperlipidemia	B-type natriuretic peptide	Interleukin 6
Congestive heart failure	C-reactive protein	Lactate
Cerebrovascular accident	D-dimer	Procalcitonin
Cancer & hematological malignancy	Ferritin	Potassium
Chronic obstructive pulmonary disease	Lactate dehydrogenase	Platelet count
Atrial fibrillation	Creatinine	
History of deep vein thrombosis	Aspartate transaminase	

Table S5. The sensitivity and specificity of different cut-off scores in the model.

Score cut-off (Total score: 10)	Sensitivity	Specificity	Positive predictive value	Negative predictive value
2	0.93	0.33	0.10	0.98
3	0.83	0.53	0.13	0.97
4	0.59	0.77	0.18	0.96
5	0.45	0.87	0.22	0.95
6	0.30	0.92	0.24	0.94

Table S6. Anticoagulation treatments received by patients in the validation cohort.

In-hospital anticoagulants	VTE (N=276)	No VTE (N=3255)	p-value
Therapeutic anticoagulation	226 (81.9%)	855 (26.3%)	<0.001
Apixaban	87 (31.5%)	291 (8.9%)	<0.001
Argatroban*	3 (1.1%)	12 (0.4%)	0.107
Bivalirudin*	0 (0%)	1 (0.03%)	1.0
Dabigatran*	0 (0%)	3 (0.09%)	1.0
Edoxaban*	0 (0%)	1 (0.03%)	1.0
Enoxaparin*	63 (22.8%)	159 (4.9%)	<0.001
Fondaparinux*	3 (1.1%)	7 (0.2%)	0.038
Heparin	173 (62.7%)	436 (13.4%)	<0.001
Rivaroxaban	16 (5.8%)	64 (2.0%)	<0.001
Warfarin	41 (14.9%)	94 (2.9%)	<0.001
Prophylactic anticoagulation	8 (2.9%)	1475 (45.3%)	<0.001
Enoxaparin*	30 (10.9%)	1637 (50.3%)	<0.001
Fondaparinux*	0 (0%)	1 (0.03%)	1.0
Rivaroxaban*	0 (0%)	5 (0.2%)	1.0
No anticoagulation	42 (15.2%)	925 (28.4%)	<0.001

*Fisher-exact tests were used for these variables; chi-square tests were used otherwise
Abbreviation: VTE, venous thromboembolism

Table S7. Anticoagulation treatments received by patients in the validation cohort.

In-hospital anticoagulants	VTE (N=182)	No VTE (N=2326)	p-value
Therapeutic anticoagulation	123 (67.6%)	668 (28.7%)	p-value
Apixaban	49 (26.9%)	140 (6.0%)	<0.001
Argatroban*	2 (1.1%)	2 (0.09%)	<0.001
Bivalirudin*	0 (0%)	1 (0.04%)	0.029
Dabigatran*	0 (0%)	1 (0.04%)	1.0
Enoxaparin	35 (19.2%)	365 (15.7%)	NA
Fondaparinux*	0 (0%)	6 (0.3%)	0.25
Heparin	103 (56.6%)	217 (9.3%)	1
Rivaroxaban*	7 (3.8%)	28 (1.2%)	<0.001
Warfarin	14 (7.7%)	47 (2.0%)	0.011
Prophylactic anticoagulation*	3 (1.6%)	708 (30.4%)	<0.001
Enoxaparin	14 (7.7%)	764 (32.8%)	<0.001
Fondaparinux*	0 (0%)	1 (0.04%)	<0.001
Rivaroxaban*	1 (0.5%)	7 (0.3%)	1
No anticoagulation	56 (30.8%)	950 (40.8%)	0.453

*Fisher-exact tests were used for these variables; chi-square tests were used otherwise
Abbreviation: VTE, venous thromboembolism
